# Incremental prognostic value of the exercise electrocardiogram in the initial assessment of patients with suspected angina: cohort study

**DOI:** 10.1136/bmj.a2240

**Published:** 2008-11-13

**Authors:** Neha Sekhri, Gene S Feder, Cornelia Junghans, Sandra Eldridge, Athavan Umaipalan, Rashmi Madhu, Harry Hemingway, Adam D Timmis

**Affiliations:** 1 Newham University Hospital, London; 2 Barts and The London Queen Mary’s School of Medicine and Dentistry, London; 3 Department of Epidemiology and Public Health, University College London Medical School, London; 4 Unit of Academic Primary Health Care, University of Bristol

## Abstract

**Objective** To determine whether resting and exercise electrocardiograms (ECGs) provide prognostic value that is incremental to that obtained from the clinical history in ambulatory patients with suspected angina attending chest pain clinics.

**Design** Multicentre cohort study.

**Setting** Rapid access chest pain clinics of six hospitals in England.

**Participants** 8176 consecutive patients with suspected angina and no previous diagnosis of coronary artery disease, all of whom had a resting ECG recorded. 4848 patients with a summary exercise ECG result recorded (positive, negative, equivocal for ischaemia) comprised the summary ECG subset of whom 1422 with more detailed exercise ECG data recorded comprised the detailed ECG subset.

**Main outcome measure** Composite of death due to coronary heart disease or non-fatal acute coronary syndrome during median follow-up of 2.46 years.

**Results** Receiver operating characteristics curves for the basic clinical assessment model alone and with the results of resting ECGs were superimposed with little difference in the C statistic. With the exercise ECGs the C statistic in the summary ECG subset increased from 0.70 (95% confidence interval 0.68 to 0.73) to 0.74 (0.71 to 0.76) and in the detailed ECG subset from 0.74 (0.70 to 0.79) to 0.78 (0.74 to 0.82). However, risk stratified cumulative probabilities of the primary end point at one year and six years for all three prognostic indices (clinical assessment only; clinical assessment plus resting ECG; clinical assessment plus resting ECG plus exercise ECG) showed only small differences at all time points and at all levels of risk.

**Conclusion** In ambulatory patients with suspected angina, basic clinical assessment encompasses nearly all the prognostic value of resting ECGs and most of the prognostic value of exercise ECGs. The limited incremental value of these widely applied tests emphasises the need for more effective methods of risk stratification in this group of patients.

## Introduction

Angina is the most common initial manifestation of coronary artery disease, with an estimated annual incidence of 2 per 100 population, higher than that for myocardial infarction and other acute coronary syndromes.^1^ Prompt assessment, early after presentation, is important to determine the likelihood of coronary artery disease and to identify those patients at risk of adverse cardiac outcomes.^2^ ^3^ For these reasons rapid access chest pain clinics were identified as a management standard within the national service framework, with the recommendation that all patients with new onset, stable chest pain should receive specialist assessment within two weeks of referral.^2^ Risk seems higher in patients with new onset angina than in those with chronic angina and extends into patients with a diagnosis of non-cardiac chest pain, who accounted for almost one third of cardiac events in a recent study.^4^ ^5^ No randomised trials of management strategies in new onset angina have been carried out, however, with most evidence derived from studies in patients with angiographic documentation of coronary artery disease or with previous myocardial infarction.^6^ ^7^ ^8^

It is generally accepted that the character of the symptoms in patients with suspected angina is central to clinical diagnosis and risk assessment.^9^ ^10^ ^11^ By convention a resting electrocardiogram (ECG) is recorded and often an exercise ECG, which is a routine investigation in 59% of the chest pain clinics in the United Kingdom^12^ and was part of the initial assessment in 76% of patients with angina in the recent Euro heart survey.^13^ Whether these investigations contribute incrementally to risk assessment in this group is unknown, as previous studies have focused on cost and feasibility rather than on prognostic value.^14^ Other studies of prognostic value have been subject to selection bias through inclusion of patients with previous myocardial infarction or angiographic documentation of coronary disease,^15^ ^16^ ^17^ ^18^ ^19^ ^20^ and many have restricted analysis to patients without symptoms.^21^ ^22^ ^23^ We studied a large cohort of ambulatory patients with recent onset of suspected angina, none of whom had been previously assessed for cardiovascular disease. We identified clinical predictors of coronary events during long term follow-up and determined whether resting ECGs and exercise ECGs provide prognostic value that is incremental to that obtained from the clinical history.

## Methods

Overall, 8176 of 10 634 consecutive patients with new onset of chest pain referred by their doctor to six chest pain clinics from 1 January 1996 to 31 December 2002 were included in this study (fig 1). Fifty one per cent were seen within 72 hours of referral and a further 46% within two weeks. We excluded patients without chest pain, patients with previously diagnosed coronary artery disease (n=858), patients with incomplete data on prespecified covariates (n=685), patients not traced by central registries (n=40),^24^ ^25^ and patients whose ethnicity was black or not specified (n=875). From among these 8176 patients (cohort), a total of 4873 (60%) who had an exercise ECG recorded were stratified into two subsets: 4848 patients with a summary test result recorded (positive, negative, equivocal for ischaemia) and 1422 with additional detailed test data recorded. These groups comprised the summary ECG subset and detailed ECG subset for exercise electrocardiography, respectively. The exercise ECG was obtained in all but 7% of patients on the day of the clinic visit.

**Figure fig1:**
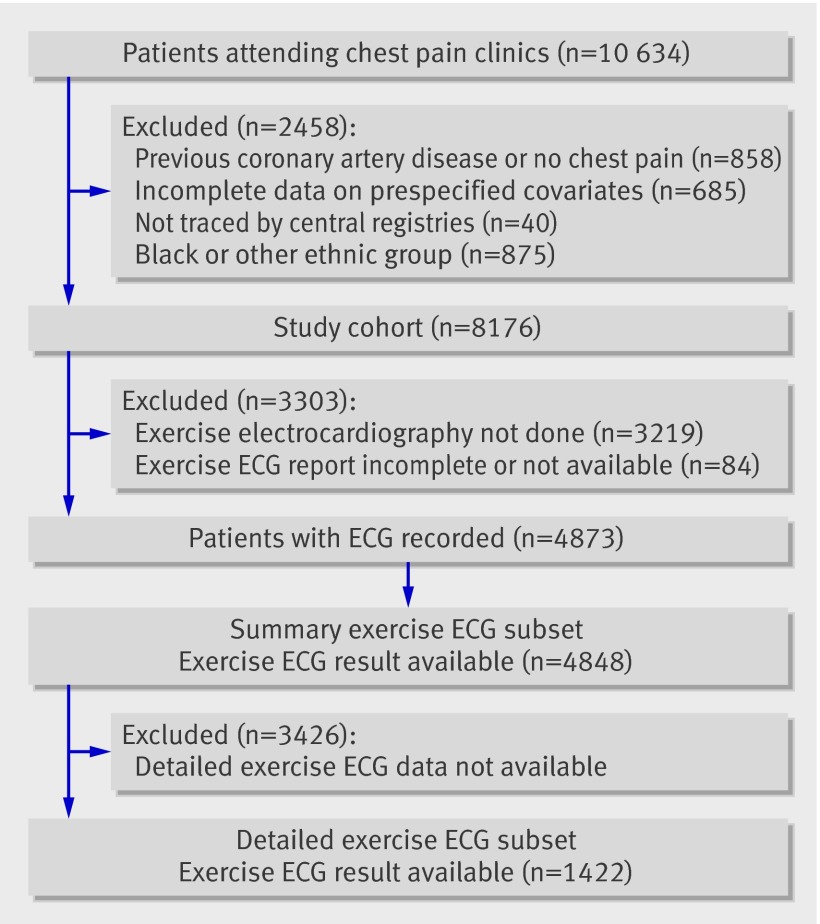
**Fig 1** Patient selection

### Data collection

We systematically recorded data in a customised database^26^ at the time of the clinic visit. Clinical data included age, sex, ethnicity, duration of symptoms, character and descriptors of chest pain, smoking status, history of hypertension, diabetes (treated using insulin or tablets), pulse rate, systolic blood pressure, treatment, and follow-up plan. At the end of the consultation we recorded the diagnosis on the basis of the clinical assessment (angina, non-cardiac chest pain, other). We obtained a resting 12 lead ECG for every patient, recorded as normal or abnormal depending on entries in the database for rhythm, conduction, regional change in ST segment or T wave, left ventricular hypertrophy, and Q waves. Treadmill stress testing was undertaken according to perceived clinical need using the Bruce protocol, with continuous monitoring of blood pressure, heart rate, and ECG up to five minutes into recovery. In the summary ECG subset only the clinicians’ assessment of ischaemia was recorded (positive, negative, or equivocal). In the detailed ECG subset, data recorded included exercise time, maximum workload, maximum heart rate, maximum blood pressure, diagnostic change in ST segment, arrhythmias, and reason for stopping (limiting symptoms, ST segment displacement of more than 1 mm 0.08 seconds after the J point, or target heart rate achieved).

### Follow-up and main outcome measure

We flagged the patients up to 23 December 2003 for mortality with the Office for National Statistics^24^ and for hospital admissions and procedures with Secondary Uses Service,^25^ with successful matching in 99.5% of cases. All outcomes were defined by codes according to the World Health Organization international classification of diseases, 10th revision.^27^ We used the primary discharge diagnosis to define events among patients undergoing hospital admission during the follow-up period.

The primary end point was a composite of death due to coronary heart disease (code I20-I25) or non-fatal acute coronary syndrome (I20-I23, 124.0, I24.8, I24.9).^27^

### Statistical analysis

We used Stata version 8.0 for all analyses. We compared the characteristics of the patients in the whole cohort with those in the ECG subsets. Data on patients who underwent coronary artery bypass grafting (code K40-K46) or percutaneous coronary intervention (K49-K50.9) were censored at the time of the revascularisation procedure because of its potential to affect prognosis. Firstly, we carried out multivariable Cox analysis for the primary end point using factors that were statistically significant at the 20% level in univariable analysis for each of three separate models: clinical model (age, sex, typicality of symptoms, heart rate, systolic blood pressure, history of hypertension, diabetes, smoking status), ECG model (QRS axis deviation, pathological Q waves, change in ST segment or T wave, left ventricular hypertrophy, bundle branch block), summary exercise ECG model (positive, negative, or equivocal), and detailed exercise ECG model (exercise time, maximum workload, percentage predicted heart rate, maximum blood pressure, reason for stopping exercise, diagnostic change in ST segment, exertional arrhythmias). We then used the covariates that remained statistically significant at the 5% level in each model to build three incremental models: basic clinical assessment, basic clinical assessment plus resting ECG, and basic clinical assessment plus resting ECG plus either summary exercise ECG or detailed exercise ECG. Then we calculated prognostic indices for each of the incremental models using the regression coefficients. To maximise power we developed the basic clinical model and the resting ECG model in the whole cohort and used the summary and detailed ECG subsets to develop the models that include data on the exercise ECG. We forced the variables from the basic clinical model and the resting ECG model into the models with data on the exercise ECGs. We plotted receiver operating characteristics curves and calculated the C statistic with confidence intervals for each prognostic index^28^ to examine the incremental prognostic value of the resting ECG and exercise ECG. Curves were plotted for all patients and for patients with intermediate probability of coronary artery disease (20-80%), based on the Diamond and Forrester algorithm, which takes into account age, sex, and typicality of chest pain.^29^ Finally, we arbitrarily split the prognostic index into thirds for risk^30^ and charted the probabilities of developing an event at one year and six years to illustrate the incremental value of the resting ECG and exercise ECG for each risk category during long term follow-up.

## Results

Participants in the whole cohort were followed-up for a median 2.46 years (interquartile range 1.61-3.92 years), in the summary ECG subset for 2.21 (1.27-3.26), and in the detailed ECG subset for 2.26 (1.51-5.18). Participants in each of the subsets had broadly similar characteristics to the cohort except for a greater proportion of patients with atypical symptoms and an intermediate probability of coronary artery disease (table 1). Angina was diagnosed in 29% of the cohort, 32% of the summary ECG subset, and 28% of the detailed ECG subset.

**Table 1 tbl1:** Clinical characteristics of cohort with suspected angina and subsets with summary exercise ECGs and detailed exercise ECGs recorded. Values are numbers (percentages) of participants unless stated otherwise

Variables	Whole cohort (n=8176)	Summary ECG subset (n=4848)	Detailed ECG subset (n=1422)
Mean (SD) age (years)	55 (13)	55 (13)	54 (11)
Character of chest pain:			
Typical	1939 (24)	1405 (29)	346 (24)
Atypical	4785 (59)	3061 (65)	989 (70)
Non-specific	1452 (18)	382 (8)	87 (6)
Male	4349 (53)	2802 (58)	824 (58)
Ethnicity:			
White	5904 (72)	3640 (75)	877 (62)
South Asian	2272 (28)	1208 (25)	545 (38)
Risk factors for cardiovascular disease:			
Current smoker	2033 (25)	1295 (27)	350 (25)
Hypertension	2783 (34)	1659 (34 )	472 (33)
Diabetes	850 (10)	531 (11)	190 (13)
Mean (SD) systolic blood pressure (mm Hg)	141 (21)	140 (20)	138 (20)
Mean (SD) heart rate (beats/min)	77 (12)	76 (12)	76 (12)
Drugs on admission*:			
Aspirin	1667 (21)	1177 (24)	213 (15)
β blocker	1199 (15)	815 (17)	183 (13)
Calcium blocker	872 (11)	492 (10)	125 (9)
Statin	887 (11)	647 (14)	126 (9)
Resting 12 lead ECG:			
Abnormal	1412 (17)	713 (15)	209 (15)
Frontal QRS axis deviation	174 (2)	99 (2)	50 (4)
Bundle branch block	266 (3)	94 (2)	34 (2)
Q waves	156 (2)	80 (2)	20 (1)
Change in ST segment or T wave	771 (9)	417 (9)	111 (8)
Left ventricular hypertrophy	263 (3)	121 (3)	27 (2)
Probability of coronary artery disease:			
<20%	1730 (21)	578 (12)	177 (13)
20-80%	4852 (59)	3148 (65)	982 (69)
>80%	1594 (20)	1122 (23)	263 (19)
Death from coronary heart disease or acute coronary syndrome	576 (7)	351 (7)	110 (8)
Death from coronary heart disease	104 (1)	51 (1)	12 (1)
Acute coronary syndrome	501 (6)	312 (6)	102 (7)
Coronary angiogram	979 (12)	708 (15)	186 (13)
PTCA	236 (3)	190 (4)	56 (4)
CABG	242 (3)	173 (4)	35 (3)
PTCA or CABG	465 (6)	354 (7)	87 (6)

PTCA=percutaneous transluminal coronary angioplasty; CABG=coronary artery bypass grafting.

*Data were not complete for cohort.

### Covariate screening and prognostic indices

Table 2 lists the covariates preselected for multivariate modelling. In the basic clinical assessment model the variables of typical chest pain, age, diabetes, and being male were independently associated with an increased risk of the composite end point of death due to coronary heart disease or non-fatal acute coronary syndrome. In the resting ECG model, variables independently associated with an increased risk of the primary end point were bundle branch block, change in ST segment or T wave, and Q waves. In the summary exercise ECG model the result was associated with the primary end point, and in the detailed exercise ECG model the variables independently associated were exercise time and change in the ST segment on exertion.

**Table 2 tbl2:** Unadjusted and adjusted hazard ratios for death from coronary heart disease or non-fatal acute coronary syndrome in three models: basic clinical assessment, resting electrocardiogram (ECG), and exercise ECG

Covariate	Univariable hazard ratio (95% CI)	P value	Adjusted hazard ratio* (95% CI)	P value
**Whole cohort (n=8167) risk of composite end point† (events=576)**				
Basic clinical assessment:				
Age (10 year increase)	1.04 (1.03 to 1.05)	<0.001	1.02 (1.02 to 1.03)	<0.001
Sex (female *v* male)	0.75 (0.64 to 0.88)	<0.001	0.76 (0.65 to 0.90)	<0.001
Typicality				
Typical *v* atypical	3.94 (3.33 to 4.67)	<0.001	3.17 (2.66 to 3.79)	<0.001
Non-specific *v* atypical	0.61 (0.45 to 0.83)		0.68 (0.50 to 0.93)	
Heart rate per 10 second increase	0.98 (0.92 to 1.05)	0.53	NA	NA
Systolic blood pressure	1.10 (1.06 to 1.14)	<0.001	1.02 (0.98 to 1.06)	0.313
Hypertension	0.71 (0.61 to 0.84)	<0.001	1.01 (0.85 to 1.21)	0.870
Diabetes	1.90 (1.55 to 2.32)	<0.001	1.48 (1.20 to 1.83)	<0.001
Current smoker	1.04 (0.86 to 1.25)	0.71	NA	NA
Resting ECG:				
Abnormal axis	2.25 (1.53 to 3.31)	<0.001	1.40 (0.94 to 2.08)	0.12
Q waves	3.73 (2.67 to 5.23)	<0.001	2.62 (1.85 to 3.70)	<0.001
Change in ST segment or T wave	2.77 (2.29 to 3.35)	<0.001	2.43 (1.98 to 2.98)	<0.001
Left ventricular hypertrophy	1.72 (1.23 to 2.40)	0.0032	1.09 (0.77 to 1.54)	0.63
Bundle branch block	2.18 (1.57 to 3.02)	<0.001	1.96 (1.40 to 2.73)	<0.001
**Summary ECG subset (n=4848), (events=351)**				
Exercise ECG:				
Positive result *v* negative result	4.58 (3.68 to 5.72)	<0.001	NA	NA
Equivocal *v* negative result	2.16 (1.48 to 3.14)			
**Detailed ECG subset (n=1422), (events=110)**				
Exercise ECG:				
Exercise time (minutes)	0.80 (0.75 to 0.86)	<0.001	0.84 (0.77 to 0.93)	0.0025
Maximum workload	0.84 (0.79 to 0.90)	<0.001	0.99 (0.93 to 1.07)	0.87
% predicted heart rate	0.99 (0.98 to 1.00)	0.0078	0.99 (0.98 to 1.00)	0.25
Maximum blood pressure	1.00 (0.99 to 1.01)	0.66	NA	NA
Reason for stopping*:				
Non-cardiac *v* target heart rate	1.95 (1.02 to 3.74)	<0.001	1.48 (0.75 to 2.93)	0.28
Cardiac *v* target heart rate	4.10 (2.15 to 7.81)		1.72 (0.85 to 3.45)	
Diagnostic change in ST segment	4.48 (3.06 to 6.55)	<0.001	3.28 (2.18 to 4.93)	<0.001
Arrhythmias	1.37 (0.82 to 2.29)	0.25	NA	NA

NA=not applicable.

*Adjusted for variables significant at 20% level in univariable analysis: non-cardiac (leg pain, fatigue, dizziness, other) and cardiac (chest pain, shortness of breath, change in ST segment, arrhythmia, abdominal pain).

†Death from coronary heart disease or non-fatal acute coronary syndrome.

Table 3 shows the results of the incremental models. In the final iterations (clinical assessment plus resting ECG plus exercise ECG) the major contributors to the risk of the primary end point were typical symptoms and abnormalities on the exercise ECG, with age, sex, and diabetes making variable additional contributions depending on whether the summary ECG subset or detailed ECG subset was analysed.

**Table 3 tbl3:** Adjusted hazard ratios for three incremental models: basic clinical assessment, basic clinical assessment plus resting electrocardiogram (ECG), and basic clinical assessment plus resting ECG plus exercise ECG

Covariate	Coefficient	Adjusted hazard ratio (95% CI)	P value
**Clinical assessment with significant variables (cohort)**			
Age (10 year increase)	0.26	1.30 (1.21 to 1.39)	<0.001
Sex (female *v* male)	−0.28	0.75 (0.64 to 0.89)	0.0008
Typicality of chest pain:			
Typical *v* atypical	1.13	3.09 (2.58 to 3.71)	<0.001
Non-cardiac *v* atypical	−0.38	0.68 (0.50 to 0.93)	
Diabetes	0.45	1.58 (1.28 to 1.94)	<0.001
**Clinical assessment plus resting ECG (cohort)**			
Age (10 year increase)	0.23	1.26 (1.17 to 1.35)	<0.001
Sex (female *v* male)	−0.27	0.76 (0.65 to 0.90)	0.0013
Typicality of chest pain:			
Typical *v* atypical	1.04	2.82 (2.34 to 3.40	<0.001
Non-cardiac *v* atypical	−0.37	0.69 (0.50 to 0.95)	
Diabetes	0.41	1.50 (1.22 to 1.86)	0.0002
Q waves	0.57	1.77 (1.24 to 2.53)	0.0037
Bundle branch block	0.30	1.36 (0.95 to 1.94)	0.1089
Change in ST segment or T wave	0.45	1.57 (1.28 to 1.94)	<0.001
Clinical assessment plus resting ECG plus summary exercise ECG*			
Age (10 year increase)	0.10	1.11 (1.00 to 1.22)	0.048
Sex (female *v* male)	−0.05	0.95 (0.76 to 1.18)	0.64
Typicality of chest pain:			
Typical *v* atypical	0.75	2.12 (1.66 to 2.71)	<0.001
Non-cardiac *v* atypical	−0.54	0.58 (0.29 to 1.19)	
Diabetes	0.36	1.44 (1.09 to 1.89)	0.0134
Q waves	0.75	2.12 (1.28 to 3.49)	0.051
Bundle branch block	−0.11	0.90 (0.40 to 2.02)	0.79
Change in ST segment or T wave	0.29	1.34 (1.01 to 1.79)	0.0078
Positive *v* negative exercise ECG	0.92	2.53 (1.95 to 3.30)	<0.001
Equivocal *v* negative exercise ECG	0.44	1.55 (1.06 to 2.28)	
Clinical assessment plus resting ECG plus detailed exercise ECG*			
Age (10 years increase)	0.03	1.03 (0.85 to 1.25)	0.76
Sex (female *v* male)	−0.59	0.55 (0.37 to 0.83)	0.0036
Typicality of chest pain:			
Typical *v* atypical	0.90	2.45 (1.62 to 3.70)	<0.001
Non-cardiac *v* atypical	−0.52	0.59 (0.14 to 2.45)	
Diabetes	0.03	1.03 (0.63 to 1.70)	0.9023
Q waves	0.49	1.64 (0.64 to 4.18)	0.3338
Bundle branch block	0.42	1.53 (0.48 to 4.89)	0.5022
Change in ST segment or T wave	0.32	1.37 (0.83 to 2.27)	0.2264
Exercise time (minutes)	−0.15	0.86 (0.79 to 0.93)	0.0005
Diagnostic change in ST segment	0.81	2.26 (1.44 to 3.53)	0.0005

*Covariates were those selected in whole cohort.

### Receiver operating characteristics curves and C statistic

In the cohort, receiver operating characteristics curves for the basic clinical assessment model alone and with iteration for the resting ECG were effectively superimposed with little or no increment in the C statistic (fig 2). With the iterations for the exercise ECGs the C statistic for the basic clinical assessment model increased in the summary ECG subset from 0.70 (95% confidence interval 0.68 to 0.73) to 0.74 (0.71 to 0.76) and in the detailed ECG subset from 0.74 (0.70 to 0.79) to 0.78 (0.74 to 0.82). When analysis was restricted to patients with an intermediate probability of coronary artery disease (20-80%), the receiver operating characteristics curves for the basic clinical assessment model alone and with iteration for the resting ECG remained effectively superimposed, reflecting poor discrimination. With the exercise ECG iterations the C statistic (95% confidence interval) for the basic clinical assessment model increased in the summary ECG subset from 0.69 (0.65 to 0.73) to 0.74 (0.70 to 0.78) and in the detailed ECG subset from 0.69 (0.62 to 0.77) to 0.76 (0.70 to 0.82).

**Figure fig2:**
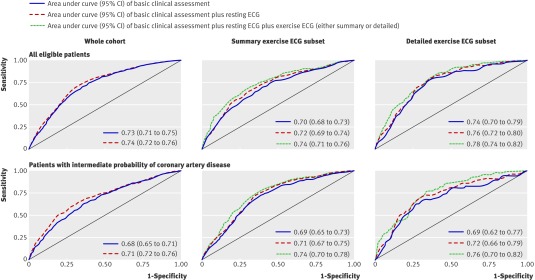
**Fig 2** Performance of prognostic indices in cohort, summary exercise electrocardiogram (ECG) subset, and detailed ECG subset in all eligible patients and in patients with intermediate probability of coronary artery disease. Prognostic indices are based on age, sex, typicality, and diabetes (basic clinical assessment); plus changes in ST segment, Q waves, and bundle branch block (basic clinical assessment plus resting ECG); plus summary ECG subset (negative, positive, equivocal), or diagnostic changes in ST segment and exercise time in detailed ECG subset

### Patient outcomes

Typical chest pain and abnormalities on the resting ECG and exercise ECG were all associated with adverse outcomes (table 4). Thus point estimates of the probability of the primary end point at three years were 16% for patients with typical chest pain, 15% for patients with an abnormal resting ECG, and 19% for patients with an abnormal exercise ECG, compared with 3%, 5%, and 9% for patients with non-specific chest pain and normal resting and exercise ECGs. However, 47% (n=166) of the events during follow-up occurred in patients with a “normal” exercise ECG, emphasising the limitations of ECGs for risk assessment. Thus in both the summary ECG and the detailed ECG subsets, risk stratified cumulative probabilities of the primary end point at one year and six years for all three prognostic indices (basic clinical assessment, basic clinical assessment plus resting ECG, basic clinical assessment plus resting ECG plus exercise ECG) showed only small differences at all time points and in all thirds of risk (table 5).

**Table 4 tbl4:** Cumulative probability of composite end point of coronary heart disease or non-fatal acute coronary syndrome at one year and six years in patients with suspected angina

Variable	% cumulative probability of end point (95% CI)	
	1 year	6 years
Character of chest pain:		
Non-specific	1 (0 to 1)	3 (2 to 4)
Atypical	2 (2 to 3)	5 (4 to 5)
Typical	8 (7 to 10)	16 (14 to 18)
Resting ECG:		
Normal	3 (2 to 3)	5 (5 to 6)
Abnormal	7 (6 to 8)	15 (13 to 17)
Exercise ECG (n=4848):		
Normal	2 (2 to 4)	9 (6 to 14)
Equivocal	4 (2 to 6)	18 (12 to 27)
Abnormal	4 (2 to 6)	19 (16 to 23)

ECG=electrocardiogram.

**Table 5 tbl5:** Cumulative probabilities of an event according to three prognostic indices in cohort and in exercise electrocardiogram (ECG) subsets

Risk groups	% cumulative probability (95% CI) at 1 year				% cumulative probability (95% CI) at 6 years		
	Clinical assessment	Clinical assessment plus resting ECG	Clinical assessment plus resting ECG plus exercise ECG*		Clinical assessment	Clinical assessment plus resting ECG	Clinical assessment plus resting ECG plus exercise ECG*
Cohort:							
Low	1 (0 to 1)	1 (0 to 1)	NA		4 (3 to 6)	4 (3 to 6)	NA
Medium	2 (2 to 3)	1 (1 to 2)	NA		10 (8 to 12)	8 (7 to 11)	NA
High	7 (6 to 8)	8 (7 to 9)	NA		25 (22 to 28)	27 (23 to 30)	NA
Summary ECG subset:							
Low	1 (1 to 2)	0 (1 to 2)	1 (1 to 2)		5 (3 to 8)	5 (3 to 8)	5 (3 to 8)
Medium	2 (2 to 3)	3 (2 to 3)	3 (2 to 3)		11 (9 to 15)	11 (8 to 14)	11 (8 to 14)
High	8 (6 to 9)	8 (6 to 9)	8 (6 to 9)		23 (20 to 27)	24 (21 to 29)	24 (21 to 27)
Detailed ECG subset:							
Low	0 (0 to 2)	0 (0 to 1)	0 (0 to 1)		5 (3 to 10)	3 (1 to 7)	3 (1 to 8)
Medium	2 (1 to 4)	2 (1 to 4)	3 (2 to 5)		8 (5 to 13)	9 (6 to 14)	9 (6 to 14)
High	8 (6 to 10)	8 (6 to 11)	7 (5 to 10)		21 (16 to 26)	22 (17 to 28)	22 (17 to 28)

NA=not applicable.

*Either incremental summary exercise ECG or detailed exercise ECG depending on subset.

## Discussion

In ambulatory patients with suspected angina, the clinical assessment embraces nearly all the prognostic information provided by the resting electrocardiogram (ECG) and exercise ECG. The limited incremental value of these widely applied non-invasive investigations extended across all thirds of risk, emphasising the importance of the clinical assessment and the need for more effective methods of risk stratification in this group of patients.

In patients with undifferentiated chest pain there is a gradient of coronary risk, greatest in those with a diagnosis of angina but extending to those with a diagnosis of non-cardiac chest pain.^5^ The importance of a careful history is widely acknowledged. Our patients with typical angina, similar to those reported in a previous study,^31^ were at higher risk of adverse outcomes than patients with atypical symptoms. Experience of non-invasive testing in patients with undifferentiated chest pain has increased in recent years, but uncertainty about its value for risk assessment remains.^14^ We found that a range of abnormalities in both the resting ECG and the exercise ECG were independently predictive of adverse events in ambulatory patients with chest pain of recent onset. It is salutary to note, however, that 47% of all events during follow-up occurred in patients with a negative exercise ECG result, emphasising the limitations of using ECGs for risk assessment, and reminding us that the demonstration of a test’s independent prognostic value does not imply clinical utility if it is not incremental to information obtained more simply from the history and examination. In this respect our findings were unequivocal, particularly for the resting ECG, which showed no incremental prognostic value above that of the clinical assessment. For the exercise ECGs, incremental prognostic value seemed somewhat greater as reflected in point estimates for the C statistic, which increased by 5.7% for the exercise ECG subset with summary results and by 5.1% for the exercise ECG subset with more detailed results. Increases were only marginally greater among patients with an intermediate probability of coronary artery disease, the group in which the exercise ECG is most useful for diagnostic purposes.^32^ How to interpret such changes in the C statistic has been debated.^33^ In clinical terms the incremental prognostic value was trivial (see table 5), with the indices that incorporated data from the exercise ECG proving no more effective than those of the basic clinical assessment in predicting adverse outcomes. Importantly, detailed analysis of variables in the exercise ECG performed little better than the summary assessment that is commonly used in clinical practice.

Previous studies of treadmill testing in chest pain clinic populations have been small and, although confirming feasibility and safety, have not been powered to test prognostic value.^14^ Before these studies, a study^15^ had developed a prognostic treadmill score in a group of patients referred for cardiac catheterisation, which has since been validated in outpatient populations.^16^ Both development and validation groups in these studies contrasted with our patients, however, being predominantly male, often with a history of myocardial infarction and other manifestations of bias from investigations owing to their selection from patients referred for cardiac catheterisation. Application of this score to patients with chest pain of recent onset in chest pain clinics therefore requires caution. Nevertheless, the incremental value of the score for predicting survival at four years in an outpatient population was modest and similar to our exercise ECG model.^16^ In another study the increment in the C statistic was similarly modest when the treadmill score was added to the clinical assessment model.^18^ More recently, prognostic scores by the ACTION trialists^34^ and Euro heart investigators^35^ have been presented, but these scores utilise data not always available at first presentation and apply to patients with chronic stable angina, many with a history of myocardial infarction and whose risk characteristics are different from the patients with suspected angina in the present study.

The methods in this study are robust and reflect the incremental value of the different statistical models rather than simple comparison of likelihood ratios. We excluded only 7% of patients (n=725) because of missing data or not being traced by central registries. This is unlikely to have affected our conclusions because outcomes in this group were not significantly different from those included in our study. Important limitations were the absence of data on lipid levels and family history, although had these been available they would probably have improved the discriminatory power of the clinical model and would not therefore have affected our conclusions. Similarly, our conclusions would not have been affected by exclusion of the 167 patients (2%) with Q waves, suggesting a history of silent infarction, because this would have further reduced the prognostic value of the resting ECG. An abnormal exercise ECG result influences decisions on revascularisation, which in turn might influence prognosis. A sensitivity analysis using coronary artery bypass grafting as part of the composite end point did not, however, change the findings. We did not capture the exact magnitude of change in the ST segment during the exercise test, but this is not a serious limitation for prognostic assessment. The presence of such change is indicative of ischaemia but may not correlate with coronary anatomy,^36^ ^37^ and agreements on changes in the ST segment can vary between observers. One of the most consistent prognostic markers in exercise testing is maximum exercise capacity,^32^ the specific variable (maximum duration of exercise, level of metabolic equivalents achieved, maximum workload, maximum heart rate, double product) used to summarise this being less important. The prognostic importance of age is not undermined by its lack of significance in the final incremental model, which merely indicates that in patients undergoing exercise testing, more prognostic weight is contributed by exercise time, the presence of change in the ST segment, and typical chest pain.

In conclusion, our study emphasises the importance of the clinical assessment for prognosis in patients with suspected angina. The data show that the need to improve risk stratification cannot be met by the resting ECG whereas the incremental value of the exercise ECG is small. Alternative tests are needed but must be developed within cohorts from chest pain clinic because prognostic value depends on the population in which the test is applied.^38^ A prerequisite of any new test should be the demonstration of its incremental value over clinical assessment if risk stratification is to be improved and the potential for chest pain clinics to reduce coronary mortality is to be fully realised.

**What is already known on this topic**

Accurate identification of patients with suspected angina at higher risk of acute coronary syndromes and death is essential to tailor management strategies
Resting and exercise electrocardiography are the most widely performed non-invasive tests in patients with suspected angina but their contribution to risk assessment is unknown
Most research has focused on newer and more costly tests for diagnosing coronary disease, with less attention to incremental value for risk assessment

**What this study adds**

Clinical assessment of patients with suspected angina embraces most of the prognostic information provided by resting ECGs and by exercise ECGs
The incremental prognostic value of detailed exercise ECG variables is little better than the summary assessment of ischaemia commonly used
More effective methods of risk stratification in this group of patients are needed

## Notes

Contributors: NS collected and analysed the data and wrote the paper. CJ and SE analysed the data. AU and RM collected the data. HH interpreted the data and wrote the paper. ADT designed the database, interpreted the data, wrote the paper, and is guarantor. All authors participated in the discussion and interpretation of the final results, contributed to the final paper, and approved the final version submitted for publication. The authors had full access to the data and take responsibility for its integrity.

Ethical approval: This study was approved by the multiregional ethics committee (/02/04/095). Permission was given by the National Patient Information Advisory Group (www.advisorybodies.doh.gov.uk/piag/) to link anonymised datasets without individual patient consent.

Provenance and peer review: Not commissioned; externally peer reviewed.

## Notes

**Cite this as: ***BMJ* 2008;337:a2240
